# Round the Hospitals

**Published:** 1921-01-01

**Authors:** 


### ROUND THE HOSPITALS.

WHAT is described as "a communal nursing scheme" has been started in Manchester, and claims to be the first of its kind in this country. No fewer than 130 approved societies sent representatives to a meeting in the Town Hall on November 30, and cordially assented to the proposals made. Each member of a society resident in the area is to pay 3d. annually, and this small tax is estimated to produce £3,000. A joint contribution of £1,500 from the Insurance and Health Committees will raise the amount to £4,500, which it is believed will provide an efficient service of well-paid nurses. Mr. Walter Davies, the Chairman of the Insurance Committee, forecasted a nursing organisation in Manchester "which would not only be the envy of every other city in the country but the basis upon which other centres would build in supplying civic nursing." The promoters are, at any rate, giving a fine example of united action in the task of securing good nurses.

Strange revelations were made by the Superintendent of Nurses, Miss Hancox, at the annual meeting of the Sheffield Queen Victoria District Nursing Association. How is it possible, she urged, to stamp out disease when housing conditions continue as at present, with, in some cases, fourteen or fifteen people living in one room? Nurses are wanted more than ever, for 90 per cent. of the working classes are nursed in their own homes. Starting the year with 271 patients, they had treated 2,372 new patients in the year. Miss Hancox appealed to the Corporation to stop the building of cinema palaces and other luxury buildings until there are enough houses to accommodate the people. As superintendent for twelve years of that branch of nurses' work, she expressed herself convinced that they are not making any headway, and the cause is to be sought in the deficiency of houses.

The Higginbotham Sick Poor Nursing Association at Glasgow disclosed an arduous year's work at the recent meeting under Sir John Reid. The number of cases treated during the year was 3,618 at a total cost of £6,168, leaving unhappily a deficit of £1,000 in the accounts. Miss Mitchell, who was appointed Superintendent last year, has been compelled to resign owing to family reasons, and cordial appreciation of her work was expressed. Her place is taken by Mrs. Beattie, first District Superintendent.

With deep regret we record the death of Dr. George Peacocke, F.R.C.P.I., Chairman of the Irish branch of the College of Nursing, at his home, 2 Fitzwilliam Square, Dublin, on Christmas morning. The call was sudden, Dr. Peacocke having been out and at work on the previous Sunday. On the day following he developed symptoms of pneumonia, and from the first the gravest fears were entertained. His untimely death (in his fifty-third year) will be deeply deplored in lay and professional circles in Dublin. The deceased held many public appointments, chief among them being physician to the Adelaide Hospital, with which he had been associated since a student, and consulting physician to King George the Fifth Military Hospital, having been recently decorated with the O.B.E. in recognition of his war services. He was also a distinguished Fellow of the Royal Academy of Medicine for Ireland. Dr. Peacocke was an ardent champion of the claims of nurses to better conditions of employment, and made no secret of his belief that they are by a long way the most suitable candidates for the position of health visitor. The deceased physician was son of the late Archbishop of Dublin. He leaves a widow and two daughters to mourn his loss.

At a meeting of the Cardiff Health Committee, last week, Alderman Davis advocated a uniform scale of salaries applicable to both health nurses and school nurses, and pleaded for consideration for the nursing profession, which is not so favourably placed as other professions and trades, its members being called upon to work at all hours and being morally denied the right to strike. His proposal was that the maxima of the four grades of nurses employed by the Corporation should be £240, £230, £220, and £210 instead of £200, £190, £180, and £170, as at present, to be reached by annual increments of £10, instead of the £5 increment paid now, with an equivalent increase for superintendent nurses. The Alderman did not get very enthusiastic support in his proposal from the other members, the only lady Councillor objecting that the scale would give the nurses more than the health inspectors, most of whom, a male member pointed out, were married men, whereas the nurses were mainly single. In the end the whole question of salaries and wages paid to Corporation employees was referred in a general way to the Salaries Committee.

The Health Committee of the Swansea Corporation has arranged to put the nursing staff of the isolation hospitals on the scale of salaries paid at the Swansea General Hospital. Subjoined is the scale: Matron, £250, rising by annual increments of £10 to £300; home sister, £80, rising by annual increments of £5 to £105; sister, £75, rising by annual increments of £5 to £100; staff nurse, £50, rising by annual increments of £5 to £75; assistant nurses, £35, rising by annual increments of £5 to £45; probationers, £20, rising by annual increments of £10 to £30.

It was a happy thought that inspired a certain lady residing near one of our large county hospitals to devote herself recently to a most worthy and valuable hobby. She has thrown open her house for the benefit of the sisters and nurses of the institution referred to, and invites them—so far as accommodation allows—to spend any free days or week-ends they like with her for the purpose of a thorough rest, the idea of their hostess being that a short time of real relaxation and change from usual surroundings is of incalculable benefit to those leading a strenuous and exacting life such as the nursing profession demands. It is hardly necessary to add that the kindly invitations are readily accepted, and prove to be the greatest boon, especially to those whose friends and homes are at some distance away.

Hospital food bills, which have soared beyond all attempts at striking a fair average per head, will shortly experience a downward tendency when bread and sugar are both easier. Times have changed indeed since the days when *The Hospital Commissariat* was written, and the cost of the quartern loaf as baked on the premises in the Marylebone Infirmary worked out at 2½d. About 12 oz. of bread a day is the ordinary average alike for patients and staff, or 5lb. a week, so that the average cost of bread at present prices amounts to £4 6s. 8d. a year per head instead of £1 1s. 8d. The difference in large establishments where these figures have to be multiplied by 1,000 is startling indeed, and the prospect of an early reduction of price in the loaf is matter for sincerest congratulation to hospital authorities.

The Medical Officer of Health for Preston advises the Health Committee of the Corporation to formulate the steps it proposes to take to replace those midwives now in practice whose numbers will naturally be reduced by age, disability, and death. Qualified women are not coming forward, owing to the expense of obtaining a qualification and of supporting themselves in practice without at any rate some assistance from the local authority. It is perfectly clear that the Corporation will have to offer really good salaries.

---

At Birmingham the breakdown of the District Nursing Society has caused much disquiet to the Insurance Committee, and the Medical Benefit Sub-Committee has earnestly protested against the proposed closing of the Moseley Road Nurses' Home. A new committee has been formed, under the name of "The Birmingham District Nursing Augmentation Fund Committee," for the purpose of raising funds to assist the societies, and a circular has been issued among others to every medical practitioner in Birmingham appealing for an annual subscription of £2 2s. It seems to us that the method adopted in Manchester of getting the members of approved societies to guarantee a small yearly sum is likely to produce more. It is universal popular support at which nursing associations should aim.

---

The Hartismere Guardians in East Anglia have lost three assistant nurses for want, so it is alleged, of paying serious attention to their just complaints. They appealed for alterations on the ground that they were underfed, that their hours on duty were too long, that insufficient leave was accorded, and that their salaries were too low. The result has been an unhesitating decision not to increase salaries, and a suggestion to the matron to allow more jam. The natural result of this contemptuous reception of complaints is a headline in the local papers, "Staff Want Jam"—and the resignation of the nurses. Another illustration of how not to govern institutions.

---

Much discussion has taken place recently in public on the unrest and discontent at Lewes Workhouse. There is a cottage for the nurses, in which they are housed in "an uncomfortable and unsatisfactory manner," the staff having been increased beyond the accommodation. The day nurses cannot get any recreation in the crowded quarters if the night nurses are to get any sleep. It was proposed that the old Board-room, now disused, should be utilised for the nurses, but one of the lady Guardians persisted in contending that it was the staff and not the quarters which required overhauling. We can hardly conceive of anything more likely to upset discipline and create a bad feeling than such obscure allusions in public to possible alterations. A nursing committee, with full powers to govern, instead of merely to make recommendations, is essential to the proper administration of all such institutions. While the internal management of the sick wards is discussed in public, unrest will continue.

The Battersea Maternity and Child-Welfare Committee have recommended the Council to appoint two health visitors at a salary of £300 a year each. Two nursing sisters at a salary of £75 and two staff nurses at £65, with board, washing, lodging, and uniform, are also to be appointed. Reckoning the value of the emoluments at not less than £100 a year, there seems to be still a large discrepancy between the salary of the health visitors and that of the nursing sisters. It would be interesting to know how many years the health visitors and the sisters devoted respectively to the acquirement of their professional knowledge.

---

Congratulations to the nursing staff of the Coventry and Warwickshire Hospital. At the monthly meeting the chairman (Mr. W. J. Wormell) reported the definite purchase of the Industrial Home in Leicester Street as a nurses' home. The matter has been under consideration for some time, but proceedings were stimulated by a rumour that others were disposed to make an offer for the property. Accordingly, a definite offer was submitted on behalf of the hospital to purchase for £5,025, and this has been accepted by the trustees. Part of the money is to be raised by means of a mortgage at 6 per cent., and £1,000 will be paid down. This will cost £240 a year, whereas to rent the premises, as at first proposed, would have cost £300 a year. The whole of the property is now owned by the hospital, and the chairman appears to be justified in considering it a good bargain.

---

The number of the disabled still in hospital, still bearing the onus of war after two years of peace, is hardly sufficiently brought before the eye of the public. Out of sight, out of mind. Yet at Christmas time the veil is partially rent asunder, and efforts are made by all concerned in their healing to invite co-operation, that some special cheer may gladden the silent sufferers. In Yorkshire alone there are no fewer than 1,268 ex-Service men undergoing remedial treatment in institutions. A large proportion of these cases are at the Beckett's Park Hospital, Leeds, and the Wharncliffe, Sheffield, under the Ministry of Pensions. The Hydro, at Bridlington, formerly run by Lady Nunburnholme, who still interests herself in it, has recently been taken over, and has sixty beds full out of a possible 100. At Wellburn Hall, Kirby Moorside, a charming convalescent home, lent by Mrs. Shaw, accommodates forty-two patients. Mrs. Shaw has offered to establish a small hutted hospital in her grounds for a similar number of patients when the hall passes again into her occupation, and this offer is now under consideration. A special institution at Harrogate, the Spa Hospital at Radlyon College, is set aside for cases of rheumatism needing baths and vaccine treatment. At all the Red Cross hospitals a small allowance was made for Christmas extras, but for the most part the gifts and Christmas fare which marked the festival in all the thirteen hospitals which receive the ex-soldiers were a tribute of public gratitude by individuals.

